# "The Hospital" Medical Book Supplement.—No. XXII

**Published:** 1909-10-16

**Authors:** 


### "THE HOSPITAL" MEDICAL BOOK SUPPLEMENT.—No. XXII.

CONTENTS.

**Medicine—**
The Open-air Treatment of Pulmonary Tuberculosis ... ... 73
Arthritis Deformans: comprising Rheumatoid Arthritis, Osteo-Arthritis, and Spondylitis Deformans ... ... ... ... ... 73
Essentials of Medicine ... ... ... ... ... ... ... 73
Blackwater Fever ... ... ... ... ... ... ... ... 74
Diathesis and Ocular Diseases ... ... ... ... ... ... 74

**Anatomy—**
Text Book of Anatomy ... ... ... ... ... ... ... 74
Anatomy, Descriptive and Applied ... ... ... ... ... 75

**Diseases of Children—**
Children in Health and Disease ... ... ... ... ... 75

**Surgery—**
Jejunal and Gastro-jejunal Ulcer ... ... ... ... ... ... 75
Pye's Surgical Handicraft ... ... ... ... ... ... ... 75
Die Krankheiten der Oberen Luftwege ... ... ... ... ... 75
Clinical Manual for the Study of Diseases of the Throat ... 75

**Miscellaneous Items—**
Guide to Promotion for Non-commissioned Officers and Men of the Royal Army Medical Corps ... ... ... ... ... 76
Pocket Medical Dictionary in Eight Languages ... ... ... 76
Dictionary of Ophthalmic Terms ... ... ... ... ... ... 76
E. Merck's Annual Report of Recent Advances in Pharmaceutical Chemistry and Therapeutics ... ... ... ... 76

### MEDICINE.

The Open-air Treatment of Pulmonary Tuberculosis. By F. W. Burton-Fanning, M.B., F.R.C.P. Pp. xii. +184. (London: Messrs. Cassell and Company, Limited. 2nd edition. 1909. Price 5s.)

This little volume is one of a series entitled "Modern Methods of Treatment." When it originally came out, the open-air treatment of consumption had perhaps fewer advocates than it has now; it might be urged that it is by this time so familiar that no handbook on the subject is required. There is nothing out of the way in the information given in the book before us, but there is this much in its favour—that Dr. Burton-Fanning insists throughout upon the possibilities of open-air treatment at home, and lays much stress upon the fact that every practitioner can do much towards the cure of consumptive cases without putting them to any great expense. Simplicity in the open-air treatment is the keynote of Dr. Burton-Fanning's little book.

Arthritis Deformans: Comprising Rheumatoid Arthritis, Osteo-Arthritis, and Spondylitis Deformans. By R. Llewellyn Jones, M.B., formerly Medical Officer of the Royal Mineral Water Hospital, Bath. Pp. xvi. and 365, with 38 full page illustrations. (Bristol: John Wright and Son, Ltd.; London: Simpkin, Marshall, Hamilton, Kent and Co., Ltd. 1909. Price 9s. 6d. net.)

Every medical man is agreed that the present classification of acute and chronic inflammations of joints is unsatisfactory. It is indeed difficult to find any large number of medical men whose classification of these affections is the same. This being so, we are bound to welcome any effort made in the direction of rendering the different types of joint diseases more distinct than they hitherto have been. We are not sure that Dr. Jones has added much to our actual knowledge of the subject, but we do think that several of the points, especially the clinical points, upon which he lays stress, will assist to make the present confusion less confusing. There can be little doubt that much of the present trouble in the classification of inflammatory joints is due to the fact that different authorities are at sixes and sevens as to precisely what they mean when they use terms like arthritis deformans, rheumatoid arthritis, osteo-arthritis, and so forth. A great advance will be made in the classification of joint lesions when clinicians have come to some agreement as to precisely what the different names they use strictly imply. We think that Dr. Jones has done much to clear the ground in this direction. Roughly speaking, he insists that arthritis deformans is merely the term to be used for any inflammatory affection of the joints which produces deformity; that is to say, alters their shape more or less permanently. He would urge that the term arthritis deformans should only be used in this loose way, and that it should never be applied to a joint disease, except until such a time as the precise nature of the latter has been decided by further examination. He then goes on to point out that if arthritis from trauma, tuberculosis, syphilis, gonorrhœa, pyæmia, and gout be excluded, the remaining joint lesions are, for the most part, divisible into two main groups—namely, first, those cases in which the outlines of the bones are not altered; and, secondly, those in which, in addition to the changes in the joints, the shape and structure of the ends of the bones are considerably altered also. There are, it is true, sub-headings for each of these main groups, according as the affection is acute, sub-acute, or chronic, or as to whether a single joint is involved, or many joints, large joints or small ones; but without our going into detail as to these, Dr. Jones makes his point very clear indeed when he says that rheumatoid arthritis is an affection distinct from osteo-arthritis, the bones not being affected in the former and being much affected in the latter. It would take us beyond the scope of this brief review were we to enter upon Dr. Jones's discussion of the neuro-trophic nature of these joint affections. We must refer the reader to the book itself. It is well worth reading, contains most useful suggestions as to treatment, and is well printed and arranged. We can commend it highly as a useful book for all practitioners, even though some may disagree with the author's personal views.

Essentials of Medicine. By Charles Phillips Emerson, M.D. (Philadelphia: J. B. Lippincott Company. 1909. Price 8s. 6d. net.)

A little physiology, a little pathology and bacteriology, a little medicine, a little hygiene: of these is composed the volume which Dr. Emerson offers to medical students, nurses, and the lay public. The American medical student, as is well known, is educated on a somewhat different system to that which prevails in this kingdom; and if, as the author asserts, he lacks perspective and an appreciation of the relative importance of many parts of his curriculum, it must be the system which is at fault. It is partly to correct this that the present work has been composed. While far from suggesting that medical education on this side is perfect, it seems to us probable that our students hardly need this particular form of guidance. As regards nurses and the public, however, we do feel that this book is a great advance on extant literature. In its readers no knowledge of physiology and pathology is assumed, and although symptoms of diseases are correctly indicated, differential diagnosis is hardly dealt with at all, and treatment is of the sketchiest. In short, Dr. Emerson's pages are likely to prove of interest and value to nurses and the public, and at the same time to bring home to the latter many of the difficulties which their medical attendants have to confront. Self-drugging and every-man-his-own-physician faddists will find nothing but discouragement in this book. We consider that the price is unnecessarily exorbitant.

Blackwater Fever. By A. G. Newell, M.D. (London: J. Bale, Sons, and Danielsson, 1909. Price, 5s. net.)

The author of this new book on blackwater fever holds very strongly that the disease is a form of malaria, or rather a complication of the malignant tertian form of malaria. He proposes, therefore, the name of bilious malignant tertian ague: this seems a quite unnecessary change, since the old name describes shortly and vividly the leading feature of the disease, and commits no one to any specific theory of its ætiology. All the same, the author is probably right in following many recent authorities on this question in his conclusion that without malarial infection there is no blackwater fever. So far Dr. Newell's views coincide with those of many others who have had extensive experience of this fever. He proceeds, however, to enunciate a theory of the exact manner in which the hæmoglobinuria arises which, we confess, we do not understand. The exposition is difficult to follow, mainly by reason of the author's peculiarly involved and obscure style, but also by his use of a large number of vague terms which he nowhere defines. The treatment he recommends is a routine dose of half to one ounce of Epsom salts to begin with, with a small dose of morphia hypodermically and hot fomentations to the stomach (*sic*) if vomiting is excessive; calomel he discountenances. He advises an interval of twenty-four hours before ordering quinine if a good deal has been lately taken; but if but little has been ingested, he gives a dose of 3 grains intramuscularly immediately after the Epsom salts, and continues this four or six hourly. Cassia beareana is also a favourite remedy with him at this stage. The most important symptoms to be treated are shock, thirst, and vomiting. We cannot honestly say that we think the problems of blackwater fever are much advanced by this monograph.

Diathesis and Ocular Diseases. By A. Maitland Ramsay, M.D., F.F.P.S., Ophthalmic Surgeon, Glasgow Royal Infirmary. (London: Baillière Tindall, and Cox. 1908. Pp. 184. 17 plates. Price 3s. 6d. net.)

In devoting a monograph entirely to the influence of various constitutional states upon certain local disorders of the eye, Dr. Ramsay calls attention to aspects of ophthalmic practice the importance of which is often neglected, even by specialists in diseases of the eye. The diatheses into which he divides mankind at large are the neurotic, the scrofulous, and the arthritic; the latter having two sub-divisions, the gouty and the rheumatic varieties. The bilious diathesis of Laycock he prefers to regard as a modification of the others than as a separate entity. In his introductory chapters are laid down briefly the signs whereby these constitutions may be distinguished, though readers are warned that mixtures of diatheses may complicate the problem of assigning an individual to one class or another. Thus in one passage we read that those who suffer from toxic amblyopia are usually of the asthenic or bilious type of arthritism, and that many are in addition markedly neurotic. The descriptions of these diatheses are not very full, nor are they to our mind convincing, though the influence of heredity in modifying the resistance of the individual to certain diseases we fully concede. But this does not satisfy the author, who makes equally short work of the suggestion lately propounded that the "scrofulous" looking patient is merely one in whom latent tuberculosis already exists, not one whose natural resistance is to begin with deficient. In the chapters which follow, Dr. Ramsay deals with inflammations of the various regions of the eye, with toxic amblyopias and retrobulbar neuritis, and with glaucoma. Here he is both sound and practical. If in places he pushes his diathetic views rather further than is necessary, at least his insistence on constitutional treatment of many ocular disorders is very valuable. Nor are strictly local remedies and forms of treatment omitted, though no details of operative technique are given. The two last chapters, on toxic amblyopias and glaucoma, are to our mind the best. The book is one which can be especially recommended to those practitioners who do their own eye-work wholly or in part; and also to students, though it is of little use for examination purposes. In recommending to British readers the work of M. Giraud, the author has borrowed from that gentleman the adjective diathesic, which he uses throughout his pages. With all respect, we would suggest that diathetic is a form superior from the point of view both of euphony and of correctness.

### ANATOMY.

Text Book of Anatomy. Edited by D. J. Cunningham, F.R.S., M.D., assisted by ten contributors. Illustrated with 936 wood engravings from original drawings. Third edition. (Mr. Henry Frowde, Oxford University Press, and Messrs. Hodder and Stoughton, Warwick Square, E.C.)

A melancholy interest attaches to this, the third edition of what has come to be known familiarly as "Cunningham's Anatomy," for it represents the last literary work that Professor Cunningham undertook before his death in June last. It is, indeed, a worthy memorial to him, and the more to be admired when we know that much of the revision was accomplished when the editor was in failing health. Taking the book as a whole and disregarding details which cannot be adequately dealt with in the brief space at our disposal, we notice a general improvement in the work which makes the book one of the premier text-books on anatomy in the English language. Numerous additions have been made, especially in the section dealing with osteology, written by Professor Arthur Thomson, in which the latest known information regarding the structure and details of individual bones has been incorporated. All the sections have been brought up to date and many new illustrations added. An attempt has also been made to introduce the Basle nomenclature in some parts, but care has been taken to avoid any confusion by providing readers with a good glossary. The index is almost a model of what such things should be, and the book has been printed and published in the excellent manner which we have come to regard as a characteristic of the united firms that are responsible for its production.

To some extent Cunningham's Anatomy challenges comparison with other text-books, and from the general practitioner's point of view it is evident that its many excellencies may not be altogether desirable. It is essentially a student's book; not indeed an elementary student's book, but primarily an anatomist's manual. The arrangement of the sections is, in our opinion, a bad one. It would be far better to give the surgical anatomy of each organ at the end of the description than to have a separate section entirely devoted to it. Again, we do not think that sufficient attention is paid to the action of muscles and joints; certainly the tabular form in which it is condensed is unattractive and, we venture to think, uninteresting. Fine features of the book are the appendices to the osteological section, and the very good description of the nervous system, in both of which special attention has been paid to morphology. These, however, are scarcely points that will appeal to the busy practitioner, who, if he needs a text-book of anatomy, needs one in which each point he desires to look up is complete in itself. For that reason we do not think "Cunningham" is *par*

*excellence* the practitioner's text-book, but there can be little question that, for the use of the student, and especially the advanced student reading for the higher qualifications, it is one of the best books on anatomy that are to be found on the market.

Anatomy, Descriptive and Applied. By Henry Gray. F.R.S. Seventeenth edition. Edited by Robert Howden, M.D., C.M. With the Notes on Applied Anatomy revised by A. J. Jex Blake, M.B., M.R.C.P., and W. Fedde Fedden, M.S., F.R.C.S. 1,032 illustrations. (London : Longmans, Green and Co., 39 Paternoster Row. Price 32s. net.)

Our old friend Gray, for the seventeenth time of printing, as good as ever and better than before! No English-speaking practitioner is ignorant of this book, which, if popularity and worth count for anything, ranks among the greatest medical works issued by any firm in these islands. So well known is it that no space need be occupied in pointing out its many excellencies or expatiating on its merits. The present issue differs from its last predecessor in that it has been completely revised, and in several parts entirely rearranged. Two hundred new engravings, many of them from original preparations and reproduced in colour, have been added, and the figures are as clear and excellent as they appeared in the sixteenth edition. Special attention has been paid to the notes on applied anatomy, which have been revised, and indeed almost re-written, by Dr. Jex Blake, so far as medical applied anatomy is concerned, and by Mr. Fedden, who deals with the surgical section. These notes now appear at the end of each section or description of each organ, and the original chapters on hernia are incorporated in the new departments on the applied anatomy of the intestine. We have no space available to allow us to go into details, much as we should like to point out new matter that appears in the various sections (especially noticeable, for instance, in the section on neurology, which is up to date in everything appertaining to the anatomy of nerves, and illustrated with particularly fine cuts showing the cutaneous and deep distributions of the various branches), and we can only express our hearty admiration for the work and congratulate the editor and revisers upon having so successfully accomplished the not very easy task of bringing the book up to date and enabling it to hold its high level against the many weighty competitors in the field. The general practitioner must have a good work on anatomy on his shelves, and he cannot do better than invest in this edition of Gray.

### SURGERY AND LARYNGOLOGY.

Jejunal and Gastro-jejunal Ulcer. By Herbert J. Paterson, F.R.C.S.Eng. (John Bale, Sons and Danielsson, Ltd., 83-91 Great Titchfield Street, London, W. 2s. 6d. net.)

We have already dealt elsewhere with the important questions raised by Mr. Paterson in this work. The author has been well advised in issuing his paper in a separate form : in the mass of matter which has accumulated since the Proceedings of the Royal Society of Medicine began so valuable a monograph would have been lost. It is somewhat a pity however that the *format* of the reprint has not been changed, for the quarto is an unwieldy size for a pamphlet.

Pye's Surgical Handicraft. Fifth edition. Revised and rewritten by W. H. Clayton-Greene, F.R.C.S. (Bristol : John Wright and Sons, Limited. 1909. 12s. 6d. net.)

Since this work was first issued in 1884 it has been through several editions, and this, the fifth, is in many ways a new book. As such it has all the excellencies of former issues, besides being thoroughly up to date and containing a large amount of new matter. The book is an epitome of general and special surgical handicraft, which we can thoroughly recommend to the practitioner who desires a concise manual of modern surgical methods. The illustrations are in every way worthy of the printed matter.

Die Krankheiten der Oberen Luftwege. Moritz Schmidt, Vierte Auflage. (Berlin : J. Springer. 1909. M. 26.)

Several things show the consideration in which this text-book has always been held ; its ready sale at a high price, the demand for it among German second-hand booksellers, and the fact that some leading British laryngologists (a body by no means averse from this form of authorship) have pronounced it the best hand-book on diseases of the nose and throat in existence. Professor Meyer's edition —the first since the author's death—shows no change in the general arrangement, although illustrations have been added, and diseases of the accessory sinuses allotted a separate chapter. The book supports those who claim the thyroid gland as part of the laryngologist's province.

Clinical Manual for the Study of Diseases of the Throat. By J. Walker Downie, M.B., F.F.P.S.G. (Glasgow : Jas. Maclehose and Sons. Second Edition, 1909.)

This is a second edition in name only, for the preceding edition has been much re-modelled and almost wholly re-written in the process of bringing it up-to-date. The many advances in all parts of throat surgery during recent years render this almost inevitable, and we congratulate the author on his determination to give the profession of his best instead of yielding to the temptation of merely tinkering with the older edition. The study of the diseases of the mouth, naso-pharynx, and pharynx, as well as of the larynx, is included in the volume. The teaching is sound and practical, and the needs of the practitioner are kept steadily in view. The illustrations, many of them in colour, are on the whole satisfactory, and enhance the usefulness of the volume. We could have wished to find on one or two points rather more decisive teaching. Thus, intubation and tracheotomy are both described in dealing with laryngeal diphtheria, but no indication is given as to which the author prefers, or of the circumstances in which one might be preferable to the other. Furthermore, there is nowhere to be found descriptions or figures of the various forms of tracheotomy tube, and the details given of the after-treatment are meagre in the extreme. The general arrangement is good, and the work can be recommended to practitioners as a convenient and reliable text-book of not too bulky a size.

### DISEASES OF CHILDREN.

Children in Health and Disease. By David Forsyth, M.D. (London : John Murray. Pp. 362. 10s. 6d. net.)

The main title of this book suggests an extensive work on the physiology and ailments of children, but a sub-title indicates that it is limited to "A Study of Child Life." It is divided into chapters on physiology, psychology, the hygiene of schools and school life, the medical aspects of school life, medical inspection and training of school children, the training of children, idiots and feebleminded children, infant mortality, the conditions of infant life, children and disease, on examining infants and children, the diagnosis of disease in children, and some points in the treatment of sick children. This summary sufficiently represents the scope of the work, and, seeing that it is printed in fairly large type and consists of only about 150,000 words, it is obvious that each subject can only be briefly considered. In a chapter of 18 pages is given a somewhat discursive summary of antenatal physiology, but it is obviously impossible in such a limited space to more than indicate some of the chief features and the problems requiring solution. It affords little information to the educated physician, and is beyond the comprehension of the ordinary layman. In 36 pages on postnatal physiology and the physiology of growth, special functions, metabolism, heat, nutrition and puberty, some useful facts are stated. The writer devotes several pages to the estimation of the calorie value of food, and its relationship to weight and surface area, in the feeding of infants. In practice this is both complicated and unnecessary, and the subject is much more suitable for discussion before a medical society than in a book of this type, which appears to have been written more for the educationist than for the medical profession. In the section on psychology simple truisms are expanded and expressed in excellent language. School life receives adequate consideration, except for the sketchy description of the dietary. This is of little practical value, for all details are omitted. The amount of protein needed is not stated, and the advantages of fruit and vegetables are ignored. The chapter on the training of children can be read with advantage by all parents, and affords suitable material for lectures to budding school masters and mistresses. That on idiots and the feeble-minded is more suitable for public guardians, school-board authorities, and members of Parliament. The study of infantile mortality is mainly an analysis of the Registrar-General's returns, and is of importance politically, socially, and economically. Passing on, we find that only four chapters, 72 pages in all, are devoted to children in disease; and they are much more suitable as an introduction to a work on diseases of children, than as part of a book interesting to the general public. Throughout, the author may be complimented on his accuracy. There is little to complain of in the statements advanced, though we disagree with the recommendation of the use of hardening lotions for the nipples, and regard eight feeds in 24 hours as too liberal a dietary for infants of from three to five months of age. It seems that the author has tried to appeal to too wide a public, chiefly the layman, and that he has thrown in a certain amount of material suitable for the profession with a view to giving his work a scientific aspect. Mothers, teachers, eugenists, publicists, and doctors will find it, like the curate's egg, "good in parts" for their special needs, but each will have to omit much that is superfluous. Except perhaps medical students and newly qualified medical practitioners, who are likely to become parents and take an active interest in public life and social questions, there is no public for whom we can regard the book as particularly appropriate.

### MISCELLANEOUS ITEMS.

Guide to Promotion for Non-commissioned Officers and Men of the Royal Army Medical Corps. Compiled by Capt. S. T. Beggs, M.B. (Gale and Polden, 2 Amen Corner, Paternoster Row, E.C., and Wellington Works, Aldershot. Second edition. 3s. 6d. net.)

We are not acquainted with the first edition of this useful little manual, but the second edition is a concise and clearly arranged book which we can heartily recommend to non-commissioned officers and men of the R.A. Medical Corps. It is fortunate indeed that in the matter of epitomes Army men are so well catered for. One of the most concentrated of books is Wolseley's "Soldier's Pocket Book," and this manual runs that celebrated compilation very close. What there is of purely professional interest—such as, for example, the section dealing with surgical instruments and splints—is admirable. The sections dealing with nursing duties are equally good. The book is well printed and of a handy size.

Pocket Medical Dictionary in Eight Languages. Edited by Dr. J. Meyer, Berlin, in collaboration with Dr. D. O'C. Finigan, M.D. Berlin, M.R.C.P.Lond., Assistant Physician, German Hospital. (London: W. Lockwood and Co., 56 Charing Cross Road, W.C.; Vienna and Berlin: Urban and Schwarzenberg. Price not stated.)

There are many polyglot dictionaries on the market but none that specially caters for the medical man with such thoroughness as this little manual. It will enable him to pilot himself through a medical article in English, French, German, Italian, Japanese, Russian, Spanish, and Hungarian, for it has eight sub-divisions in less than eight hundred pages. Dr. Finigan remarks in the preface that the book is intended to minimise the difficulties arising from the use of an unfamiliar language "as with its aid a medical man speaking any one of the eight languages may converse in the other seven." We are not quite as enthusiastic as all that, for we tried, with its aid, to explain to a Japanese student the latest theories on the function of the basal ganglia, and we must confess that he informed us, at the conclusion of the speech, that he had very hazy notions as to what we had been talking about. Nevertheless it is an extremely useful, well got up, exceedingly well printed and arranged little book, which we cordially welcome and commend to the favourable consideration of every medical man. There are a few errors in the English, but these are obvious misprints. Infant's foot is not *Kindermehl* in German, but infant's food might be so translated. *Betriebsunfall* again is not always a factory accident. The French, Italian, and Spanish parts also contain a few misprints which should be corrected in the next edition.

Dictionary of Ophthalmic Terms. By Edward Magennis, M.D., D.P.H. (Bristol: J. Wright and Co. Price 2s. 6d. net.)

A small and useful reference book for busy practitioners, giving a concise definition and explanation of all the terms ordinarily employed in eye work. In a supplement are to be found many useful hints, which, although given in tabloid form, are clear, practical and good. The "Points worth remembering" at the end are especially so.

E. Merck's Annual Report of Recent Advances in Pharmaceutical Chemistry and Therapeutics. Vol. XXII. (Darmstadt: E. Merck.)

This little volume is a mine of information on the newer drugs, vaccines, and sera. Every note is interestingly written, and full references are given. The indexing has been specially well done, and as a work of reference "Merck's Annual" is almost indispensable to the modern practitioner who desires to be up-to-date. The price of the book is 1s. 6d., but it is forwarded free to medical men on application to the London offices of the firm, 16 Jewry Street, E.C.

